# Linc01014 regulates gefitinib resistance in oesophagus cancer via EGFR‐PI3K‐AKT‐mTOR signalling pathway

**DOI:** 10.1111/jcmm.14860

**Published:** 2019-11-30

**Authors:** Xiao Fu, Guanghui Cui, Shuaishuai Liu, Song Zhao

**Affiliations:** ^1^ Department of Thoracic Surgery The First Affliated Hospital of Zhengzhou University Zhengzhou China

**Keywords:** EGFR‐PI3K‐AKT‐mTOR signalling pathway, gefitinib resistance, linc01014, oesophagus cancer

## Abstract

This study aimed to explore the underlying mechanism of linc01014 in oesophagus cancer gefitinib resistance. Gefitinib‐resistant oesophagus squamous cell carcinoma (ESCC gefitinibR) cell lines were constructed by using different gefitinib treatment in FLO‐1, KYAE‐1, TE‐8 and TE‐5 cell lines and confirmed by MTS50 and proliferation assays. Expression of linc01014 was overexpressed/silenced in FLO‐1 cells followed by gefitinib treatment, and then, the apoptosis‐associated markers Bax and Bcl‐2, and PI3KCA in PI3K signalling pathway were determined using Western blotting. MST50 and morphology analyses showed that ESCC gefitinibR cell lines presented obvious gefitinib resistance than their parental ESCC cell lines. ESCC gefitinibR cell lines showed significantly higher proliferation abilities than their parental ESCC cell lines after treating with gefitinib. Overexpression of linc01014 significantly inhibited the apoptosis of FLO‐1 cells induced by gefitinib and silencing linc01014 obviously promoted the apoptosis of FLO‐1 cells induced by gefitinib. Silencing linc01014 could significantly increase the gefitinib chemotherapy sensitivity of oesophagus cancer via PI3K‐AKT‐mTOR signalling pathway.

**Highlights**

- ESCC gefitinib‐resistant cell lines were successfully constructed.
- Overexpression of linc01014 significantly decreased the apoptosis of FLO‐1 cells induced by gefitinib.
- Silencing linc01014 obviously promoted the apoptosis of FLO‐1 cells induced by gefitinib.

## 1 INTRODUCTION

Oesophageal cancer is one of the most leading cause of mortality cancer worldwide, and oesophageal squamous cell carcinoma (ESCC) is the main type for oesophageal cancer arising from oesophageal epithelial cells.^1^, ^2^ The recent epidemiology showed that oesophageal cancer is one of the five most common cancers in China, especially in middle area of China, and attributed for about 1 396 000 people mortalities.^3^ Despite the multiple efforts made in recent years, treatments for oesophageal cancer are still not satisfied and chemotherapy resistance has becoming a new challenge for its treatment. Therefore, it is important to reveal the mechanism of ESCC and improve the therapeutic effect of ESCC.

Epidermal growth factor receptor (EGFR), a transmembrane tyrosine kinase, plays critical role in several solid cancers and its chemotherapy resistance regulation.^4^ It is reported that about 40%‐70% of patients with ESCC have presented with high levels of EGFR expression^5^, ^6^ and inhibiting the activity of EGFR might be promising to improve the outcome of patients with ESCC. Gefitinib is a well‐known orally active, reversible and selective EGFR inhibitor applied for ESCC treatment in clinic.^7^, ^8^ However, the resistance of gefitinib has become a new challenge for ESCC treatment with unclear mechanism.^9^

Long non‐coding RNAs (lncRNAs) is a class of transcripts without coding capacity at a length of >200 nt and has reported to play critical roles in the pathogenesis and drug resistance of ESCC.^10^, ^11^ LncRNA CCAT2 is associated with poor prognosis of ESCC.^12^ High expression of lncRNA TUG1 is identified to be associated with chemotherapy resistance and poor prognosis of patients with ESCC.^13^ Kang et al^14^ have revealed that lncRNA PART1 acts as a ceRNA to induce gefitinib resistance in ESCC via miR‐129/Bcl‐2 pathway. Hence, it is significant to reveal the mechanisms of lncRNAs in regulating ESCC gefitinib resistance.

In this study, the role of lncRNA linc01014 involved in gefitinib resistance was explored in ESCC cell lines. According to these investigations, we hope to provide some new insights in solving gefitinib resistance in ESCC and improving the prognosis of patients with ESSC.

## 2 MATERIALS AND METHODS

### 2.1 Cell culture

Human ESCC cell lines KYAE‐1, TE8, TE5 and FLO‐1 were purchased from the Chinese Type Culture Collection, Chinese Academy of Sciences (Shanghai, China). All cell lines were cultured in PRIM 1640 medium (Gibco) supplemented with 10% fetal bovine serum (FBS, Gibco) and 100 U/mL penicillin/streptomycin (Sigma) at 37°C in a humidified incubator with 5% CO_2_ in atmosphere. Cells were transfected with linc01014 mimic, si‐linc01014 or negative control (NC) using lipofectamine 2000 (Invitrogen) according to manufacturer's protocol.

### 2.2 Construction of ESCC cell lines with gefitinib resistance

Gefitinib‐resistant cell lines of KYAE‐1, TE8, TE5 and FLO‐1 were constructed via repeatedly subcultured with an increasing concentration of gefitinib (Sigma) starting from 0.1 μM/mL to final concentration of 10.2 μM/mL. Finally, the gefitinib‐resistant cell lines were collected and used for the following investigations.

### 2.3 Cell proliferation assay

The cytotoxic effect of gefitinib on ESCC cell lines and the proliferation abilities of these cell lines under different treatments were detected by the MTS ([3‐(4,5‐Dimethylthiazol‐2‐yl)‐5‐(3‐carboxymethoxyphenyl)‐2‐(4‐sulphopheny)‐2H‐tetrazolium]) assay using CellTiter‐96‐AQueous One Solution Cell Proliferation Assay reagent (Promega). Briefly, cells were seeded in 96‐well plates at a density of 5 × 10^3^/well and maintained in PRIM 1640 medium contained with 10% FBS overnight. Then, cells were treated with gefitinib in dimethyl sulphoxide (DMSO, Sigma) at concentrations of 0, 1, 10, 20 and 30 μmol/L for 48 hours. Concentration of gefitinib that caused 50% inhibition on the MTS activity (MTS_50_) was determined as previously reported. For proliferation detection, cells were seeded in 96‐well plates at a density of 1 × 10^3^/well and maintained in PRIM 1640 medium contained with 10% FBS overnight. Then, cells were treated with MTS_50_ determined gefitinib concentration for 0, 48 and 72 hours. Subsequently, cell proliferation was determined. Each experiment was performed in triplicate, and average value was calculated as the results.

### 2.4 Morphology study

Morphology of cells after treating with or without gefitinib was pictured under light microscope (Olympus CKX41) at 24, 48 and 72 hours at 200× magnification. The morphological appearance of cells was compared at each time‐point.

### 2.5 Quantitative real‐time PCR

After treatment, total RNA in cells was isolated using TRIzol reagent (Takara) according to manufacturer's protocol. cDNA was synthesized using the GoScript™ Reverse Transcription System (Promega) according to manufacturer's instruction. Then, the expression of linc01014 was determined using SYBR Green Master Mix (Applied Biosystems) with a total of 2 μg cDNA as a template on a BioRad CFX96 Sequence Detection System (Bio‐Rad company). Relative expression of linc01014 was analysed using 2^−ΔΔCt^ method with GAPDH as the internal control. All premier sequences were purchased from RiBio (Guangzhou, China).

### 2.6 Western blotting

After treatment, cells were lysated with RIPA lysis buffer (Millipore) supplemented with protease inhibitors (Sigma). Protein concentrations were measured using BCA method (Pierce) according to the manufacturer's protocol (Beyotime). Then, proteins were boiled with loading buffer and subjected for 10% SDS electrophoresis followed by transferring to polyvinylidene fluoride membranes (Millipore). Subsequently, membranes were blocked with 5% milk at room temperature for 1 hour. Following this, membranes were incubated with a 1:1000 solution of primary antibodies (anti‐PI3KCA, anti‐Bcl‐2, anti‐Bax and anti‐β‐actin, Cell Signaling Technology) at 4°C overnight. Then, membranes were incubated with the secondary antibody at room temperature for 1 hour and visualized using ECL method (Pierce).

### 2.7 Statistical analyses

SPSS 19.0 (SPSS Incorporation) was used to perform statistical analyses in this study. Data in this study were presented with mean ± standard deviation (SD). Comparisons among groups were analysed using Student's *t* test or one‐way ANOVA analysis. *P* < .05 was considered statistically significant.

## 3 RESULTS

### 3.1 Evaluation for gefitinib‐resistant cell lines

**Figure 1:**
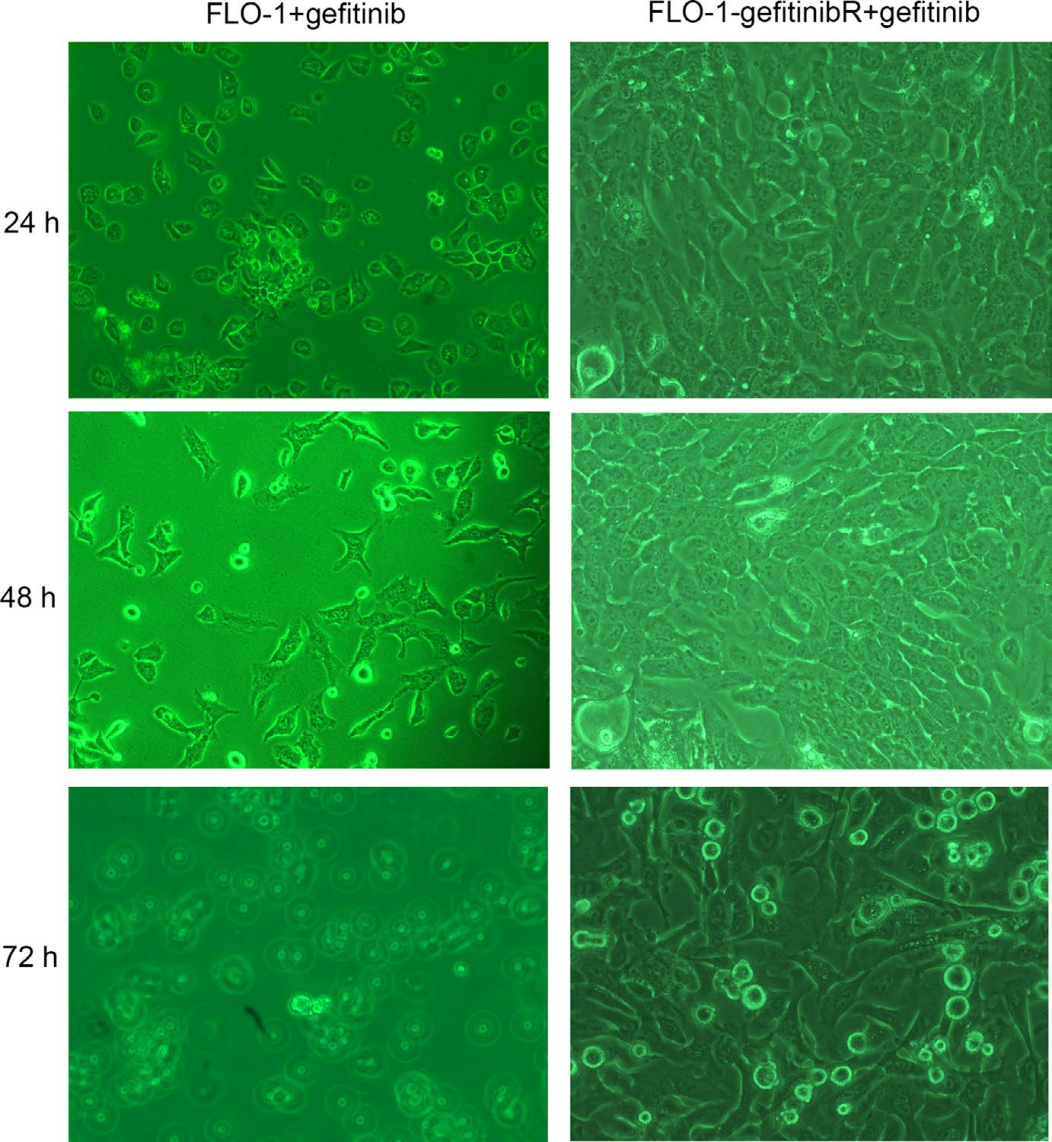
MTS50 and morphology of ESCC gefitinib‐resistant cell lines

The sensitivity of ESCC and ESCC gefitinibR cells to gefitinib was determined by MTS viability assay and cell morphology. The MTS assay showed that FLO‐1‐gefitinibR cell lines showed remarkably higher relative MTS activity than FLO‐1 cells at concentration of 0.10‐10.0 μmol/L, indicating that FLO‐1‐gefitinibR cell lines presented a higher gefitinib resistance that FLO‐1 cells (Figure 1A). According to these, the KYAE‐1‐gefitinibR, TE8‐gefitinibR and TE5‐gefitinibR cell lines were successfully constructed (Figure 1A). When FLO‐1 and FLO‐1‐gefitinibR cells were cultured in the medium with 10 μmol/L gefitinib, FLO‐1 cells presented a shrank and rounded‐up morphology and increased detachment after 48‐hours culturing, but FLO‐1‐gefitinibR cells showed increase in confluence from 0 to 48 hours and a slight detachment at 72 hours, indicating that FLO‐1‐gefitinibR cells presented a higher gefitinib resistance that FLO‐1 cells (Figure 1B).

### 3.2 ESCC gefitinibR cell lines presented higher proliferation than ESCC

**Figure 2:**
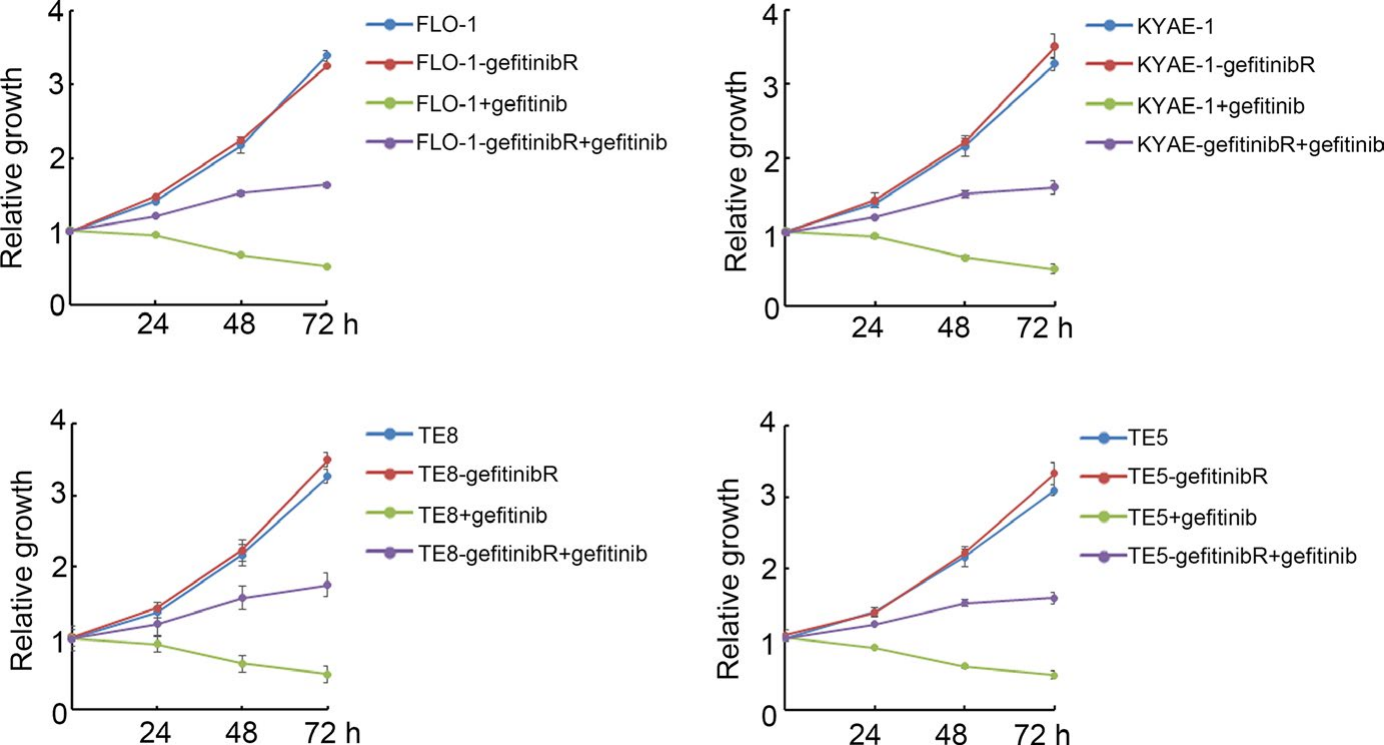
Proliferation of ESCC gefitinib‐resistant cell lines

To further confirm the property of gefitinibR cells, the proliferation of ESCC and ESCC gefitinibR cells were determined after treated with 10μM of gefitinib. The results showed that the proliferation of ESCC cell lines and ESCC gefitinib cell lines were significantly inhibited after treating with gefitinib, while the inhibitive extent in FLO‐1, KYAE‐1, TE8 and TE5 cells were significantly higher than that in FLO‐1‐gefitinibR, KYAE‐1‐gefitinibR, TE8‐gefitinibR and TE5‐gefitinibR cells, indicating that ESCC gefitinibR cells showed more gefitinib resistance than their parental ESSC cells (Figure 2).

### 3.3 Confirmation the expression of linc01014 in FLO‐1 cells

**Figure 3:**
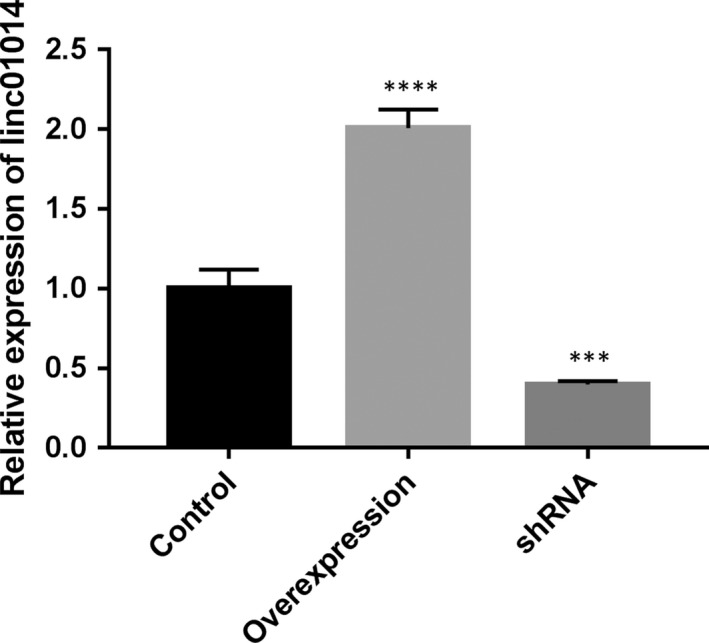
mRNA expression of linc01014 in FLO‐1 cells. Compared with the negative control (NC) group, **P < .01

To further reveal the mechanism of gefitinib resistance, the expression of linc01014 was altered in FLO‐1 cells and assessed using RT‐qPCR. The result showed that linc01014 was significantly decreased/increased in FLO‐1 cells (Figure 3), indicating that these cell lines could be used for the following investigations.

### 3.4 Overexpression of linc01014 promotes the apoptosis of FLO‐1 cells induced by gefitinib

**Figure 4:**
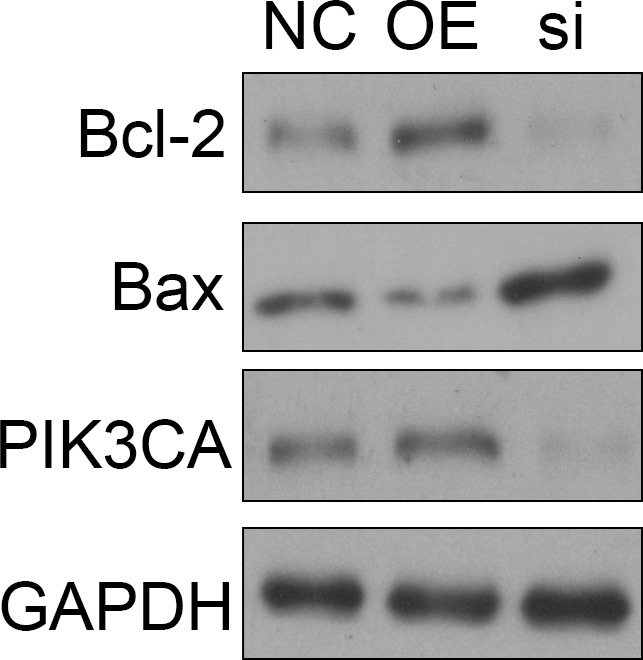
Expression of PI3KCA, Bax and Bcl‐2 in FLO‐1 cells with different expression of linc01014 and treated with gefitinib

To further reveal the function of linc01014, gefitinib (5 μm) was used to treat FLO‐1 cells after altering the expression of linc01014. The result showed that overexpression of linc01014 could significantly increase the expression of Bcl‐2 and PI3CA, but decrease the expression of Bax, indicating that linc01014 could inhibit the apoptosis of FLO‐1 cells induced by gefitinib. However, silencing the expression of linc01014 could significantly increase the expression of Bax, but decrease the expression of Bcl‐2 and PI3KCA, indicating that silencing linc01014 could promote the apoptosis of FLO‐1 cells induced by gefitinib (Figure 4).

## 4 DISCUSSION

In recent decades, several chemotherapy drugs have been explored and applied for cancer therapy, but drug resistance is becoming a new challenge for clinical therapy, which is one of the major cause for failure treatment in clinic.^15^ Although many efforts have been made, the mechanism of drug resistance remains unclear and need to be further explored. Along with the research of lncRNA, the regulatory mechanism of lncRNA in cancer provides us a new insight to understand the mechanism of drug resistance.^16^

Gefitinib is a common chemotherapy choice for patients with ESSC.^17^ The previous study has revealed that gefitinib could sensitize mutations in ESCC to chemotherapy treatment.^18^ Mechanism research has identified that gefitinib could sensitize mutation in ESCC cell line KYSE450 via EGFR.^19^ However, a phase II study about gefitinib in treating ESCC showed that although the overall response rate could reach 90%, the complete response was only 40% and median progression‐free survival was 7.0 months,^7^ suggesting there might be a large room to improve this treatment.

The previous studies revealed that PI3K/AKT/mTOR signalling pathway play crucial role in the pathogenesis of ESCC.^20^, ^21^ PIK3CA is a subunit of PI3K and often responsible for the activity of PI3K/Akt/mTOR signalling pathway.^22^ Mutation of PI3KCA could cause neoplastic transformation and accelerate cancer progression.^23^, ^24^ Shigaki et al^25^ have demonstrated that PIK3CA mutation is significantly associated with a favourable prognosis of patients with ESCC after curative resected treatment. Lin et al^26^ have identified that PI3KCA mutation and PI3K overexpression are significantly correlated with the age, tumour staging and clinical characteristics of patients with ESCC. PIK3CA mutation and overexpression are also considered to be the prognostic markers for Chinese ESCC patients.^27^ In addition, EGFR mutant actives PI3K/AKT signalling pathway via ERBB3 and inhibiting the ERBB3/PI3K/AKT signalling pathway is essential for gefitinib anti‐cancer effect.^28^ These findings suggested that modulating the PI3K/AKT/mTOR signalling pathway might alter the anti‐effect of gefitinib in treating ESCC.

Linc01014 is newly identified PIK3CA corresponding lncRNA (http://www.noncode.org/show_rna.php?xml:id=NONHSAT093370%26version=2%26utd=1#), indicating that linc01014 might play a potential role in regulating the PI3K/AKT/mTOR signalling pathway via PIK3CA. In the current study, we have identified that overexpression of linc01014 could significantly increase the expression of PIK3CA and Bcl‐2, but decrease the expression of Bax. In addition, inhibiting the expression of linc01014 could obviously inhibit the expression of PIK3CA and Bcl‐2, but induce the expression of Bax after treated with gefitinib. These findings suggested that linc01014 could significantly regulate the activity of PI3K/Akt/mTOR signalling pathway via regulating the expression of PIK3CA to altering the sensitivity of ESCC cells to gefitinib.

There were still some limitations in this study. First, because of the limited clinical samples, the expression of linc0104 in ESCC and gefitinib‐resistant ESCC tissue samples was not explored, which might impair the significance of our study. Moreover, lncRNAs have many regulatory mechanisms to modulate the pathogenesis of disease, but because of the shortage of research funds, the exact mechanism of linc0104 in regulating gefitinib resistance in ESCC was not further explored. A deepened exploration about linc0104 in gefitinib resistance is still required in the further.

In conclusion, linc0104 might play a critical role in the pathogenesis of ESCC. Silencing of linc01014 could significantly promote the sensitivity of ESCC cell lines to gefitinib and increasing the apoptosis of ESCC cells via PI3K/AKT/mTOR signalling pathway. These finding might be of importance in understanding and treatment of ESCC gefitinib resistance in clinic.

## CONFLICT OF INTEREST

All of the authors have no conflict of interest in this research.

## AUTHOR CONTRIBUTIONS

Each author has made an important scientific contribution to the study and has assisted with the drafting or revising of the manuscript.

## CONSENT TO PUBLISH

All of the authors have consented to publish this research.

## Data Availability

The data used to support the findings of this study are included in the article.

## References

[1] Torre LA , Bray F , Siegel RL , Ferlay J , Lortet‐Tieulent J , Jemal A . Global cancer statistics, 2012. CA Cancer J Clin. 2015;65(2):87‐108. 25651787 10.3322/caac.21262

[2] Dong S , Zhang P , Liang S , Wang S , Sun P , Wang Y . The role of the retinoblastoma protein‐interacting zinc finger gene 1 tumor suppressor gene in human esophageal squamous cell carcinoma cells. Oncol Lett. 2013;6(6):1656‐1662. 24260060 10.3892/ol.2013.1608 PMC3833985

[3] Chen WQ , Li H , Sun KX , et al. Report of Cancer Incidence and Mortality in China, 2014. Zhonghua zhong liu za zhi. 2018;40(1):5‐13. 29365411 10.3760/cma.j.issn.0253-3766.2018.01.002

[4] Holohan C , Van Schaeybroeck S , Longley DB , Johnston PG . Cancer drug resistance: an evolving paradigm. Nat Rev Cancer. 2013;13(10):714‐726. 24060863 10.1038/nrc3599

[5] Hara F , Aoe M , Doihara H , et al. Antitumor effect of gefitinib (‘Iressa’) on esophageal squamous cell carcinoma cell lines in vitro and in vivo. Cancer Lett. 2005;226(1):37‐47. 16004931 10.1016/j.canlet.2004.12.025

[6] Iihara K , Shiozaki H , Tahara H , et al. Prognostic significance of transforming growth factor‐α in human esophageal carcinoma implication for the autocrine proliferation. Cancer. 1993;71(10):2902‐2909. 8490817 10.1002/1097-0142(19930515)71:10<2902::aid-cncr2820711004>3.0.co;2-j

[7] Xu Y , Zheng Y , Sun X , et al. Concurrent radiotherapy with gefitinib in elderly patients with esophageal squamous cell carcinoma: preliminary results of a phase II study. Oncotarget. 2015;6(35):38429‐38439. 26392415 10.18632/oncotarget.5193 PMC4742011

[8] Petty R , Dahle‐Smith A , Stevenson DAJ , et al. Gefitinib and epidermal growth factor receptor gene copy number aberrations in esophageal cancer. J Clin Oncol. 2017;35(20):2279‐2287. 28537764 10.1200/JCO.2016.70.3934

[9] Xu Y , Peng Z , Li Z , et al. Expression and clinical significance of c‐Met in advanced esophageal squamous cell carcinoma. BMC Cancer. 2015;15(1):6. 25588551 10.1186/s12885-014-1001-3 PMC4307685

[10] Wang H , Guan Z , He K , Qian J , Cao J , Teng L . LncRNA UCA1 in anti‐cancer drug resistance. Oncotarget. 2017;8(38):64638‐64650. 28969100 10.18632/oncotarget.18344 PMC5610032

[11] Huarte M . The emerging role of lncRNAs in cancer. Nat Med. 2015;21:1253. 26540387 10.1038/nm.3981

[12] Zhang X , Xu Y , He C , et al. Elevated expression of CCAT2 is associated with poor prognosis in esophageal squamous cell carcinoma. J Surg Oncol. 2015;111(7):834‐839. 25919911 10.1002/jso.23888

[13] Jiang L , Wang W , Li G , et al. High TUG1 expression is associated with chemotherapy resistance and poor prognosis in esophageal squamous cell carcinoma. Cancer Chemother Pharmacol. 2016;78(2):1‐7. 27329359 10.1007/s00280-016-3066-y

[14] Kang M , Ren M , Li Y , Fu Y , Deng M , Li C . Exosome‐mediated transfer of lncRNA PART1 induces gefitinib resistance in esophageal squamous cell carcinoma via functioning as a competing endogenous RNA. J Exp Clin Cancer Res. 2018;37(1):171. 30049286 10.1186/s13046-018-0845-9 PMC6063009

[15] Pan S‐T , Li Z‐L , He Z‐X , Qiu J‐X , Zhou S‐F . Molecular mechanisms for tumour resistance to chemotherapy. Clin Exp Pharmacol Physiol. 2016;43(8):723‐737. 27097837 10.1111/1440-1681.12581

[16] Majidinia M , Yousefi B . Long non‐coding RNAs in cancer drug resistance development. DNA Repair. 2016;45:25‐33. 27427176 10.1016/j.dnarep.2016.06.003

[17] Janmaat ML , Gallegos‐Ruiz MI , Rodriguez JA , et al. Predictive factors for outcome in a phase II study of gefitinib in second‐line treatment of advanced esophageal cancer patients. J Clin Oncol. 2006;24(10):1612‐1619. 16575012 10.1200/JCO.2005.03.4900

[18] Guo M , Liu S , Lu F . Gefitinib‐sensitizing mutations in esophageal carcinoma. N Engl J Med. 2006;354(20):2193‐2194. 16707764 10.1056/NEJMc052698

[19] Guo M , Liu S , Herman JG , Zhang H , Lu F . Gefitinib‐sensitizing mutation in esophageal carcinoma cell line Kyse450. Cancer Biol Ther. 2006;5(2):152‐155. 16357520 10.4161/cbt.5.2.2318

[20] Li H , Gao Q , Guo L , Lu SH . The PTEN/PI3K/Akt pathway regulates stem‐like cells in primary esophageal carcinoma cells. Cancer Biol Ther. 2011;11(11):950‐958. 21467840 10.4161/cbt.11.11.15531

[21] Zhang H‐B , Lu P , Guo Q‐Y , Zhang Z‐H , Meng X‐Y . Baicalein induces apoptosis in esophageal squamous cell carcinoma cells through modulation of the PI3K/Akt pathway. Oncol Lett. 2013;5(2):722‐728. 23420294 10.3892/ol.2012.1069 PMC3572959

[22] Samuels Y , Wang Z , Bardelli A , et al. High frequency of mutations of the PIK3CA gene in human cancers. Science. 2004;304:554. 15016963 10.1126/science.1096502

[23] Kang S , Bader AG , Vogt PK . Phosphatidylinositol 3‐kinase mutations identified in human cancer are oncogenic. Proc Natl Acad Sci USA. 2005;102(3):802‐807. 15647370 10.1073/pnas.0408864102 PMC545580

[24] Engelman JA . Targeting PI3K signalling in cancer: opportunities, challenges and limitations. Nat Rev Cancer. 2009;9(8):550‐562. 19629070 10.1038/nrc2664

[25] Shigaki H , Baba Y , Watanabe M , et al. *PIK3CA* mutation is associated with a favorable prognosis among patients with curatively resected esophageal squamous cell carcinoma. Clin Cancer Res. 2013;19(9):2451‐2459. 23532889 10.1158/1078-0432.CCR-12-3559

[26] Lin J‐W , Li X , Qiu ML , Luo RG , Lin JB , Liu B . PI3K overexpression and PIK3CA mutations are associated with age, tumor staging, and other clinical characteristics in chinese patients with esophageal squamous cell carcinoma. Genetic Testing Mol Biomarkers. 2017;21(4):236‐241. 10.1089/gtmb.2016.0316 28384037

[27] Wang L , Shan L , Zhang S , et al. PIK3CA gene mutations and overexpression: implications for prognostic biomarker and therapeutic target in chinese esophageal squamous cell carcinoma. PLoS ONE. 2014;9(7):e103021. 25054828 10.1371/journal.pone.0103021 PMC4108430

[28] Engelman JA , Zejnullahu K , Mitsudomi T , et al. MET amplification leads to gefitinib resistance in lung cancer by activating ERBB3 signaling. Science. 2007;316:1039‐1043. 17463250 10.1126/science.1141478

